# Correlation Between Gut Microbiota and Plasma Metabolites in a Mouse Model for Post-Traumatic Stress Disorder

**DOI:** 10.3390/metabo16040222

**Published:** 2026-03-28

**Authors:** Daxue Zhou, Youying Huang, Fei Li, Qin Liu, Xiaoyang Wang, Quanfang Wei, Jiajia Chen, Zhilong Liu, Yi Huang

**Affiliations:** 1 Biomedical Analysis Center, Army Medical University, Chongqing 400038, China; zhoudaxue2008@126.com (D.Z.); lf112.2004@163.com (F.L.);; 2 Department of Tropical Medicine, College of Military Preventive Medicine, Army Medical University, Chongqing 400038, China

**Keywords:** post-traumatic stress disorder, gut microbiome, plasma metabolome, 16S rRNA sequencing, UHPLC-MS/MS

## Abstract

Background: The gut microbiota and plasma metabolites have been shown to contribute to the etiology of post-traumatic stress disorder (PTSD). The relationship between the gut microbiome and plasma metabolome in PTSD is poorly understood. This study aims to integrate the gut microbiome data and plasma metabolome data to elucidate microbial–metabolite associations specific for PTSD in a mouse model. Methods: A PTSD mouse model was induced by single prolonged stress and electric foot shock (SPS&S). We sequenced the gut microbiota composition by 16S rRNA gene sequencing and used ultra-high performance liquid chromatography–tandem mass spectrometry (UHPLC-MS/MS) for the plasma metabolomic profiling to explore the association between the gut microbiota and the plasma metabolites in mice with PTSD. Results: The PTSD mice exhibited robust anxiety-like behaviors, significantly elevated plasma IL-1β and TNF-α, and profound gut dysbiosis characterized by a marked depletion of Muribaculaceae and Akkermansia and expansion of the Lachnospiraceae_NK4A136_group. The plasma metabolomics identified 24 significantly dysregulated metabolites, including upregulated L-arginine, palmitic acid, and oleic acid, and downregulated uridine. The pathway enrichment analysis revealed coordinated perturbations in arginine biosynthesis, pyrimidine metabolism, unsaturated fatty acid biosynthesis, and glycerophospholipid metabolism. Critically, genus-level correlation analysis uncovered biologically coherent associations. The Muribaculaceae abundance showed strong negative correlations with L-arginine and palmitic acid and positive correlations with L-glutamine and thymidine. Conclusions: This study provides an exploratory investigation into the association network between the gut microbiota and the plasma metabolites in a PTSD mouse model, offering preliminary insights into potential microbe–metabolite interactions in PTSD.

## 1. Introduction

Post-traumatic stress disorder (PTSD) is a psychiatric disorder characterized by the delayed emergence and chronic course following an exposure to traumatic events. Its core clinical manifestation is involuntary and sensory-intense as the sufferer re-experiences the traumatic event [1,2]. PTSD frequently co-occurs with other psychiatric disorders and is associated with an elevated risk for multiple physical conditions, including gastrointestinal disorders [3,4,5], immune dysregulation, and metabolic syndrome [6,7]. Notably, during the 2019 COVID-19 pandemic, a multinational epidemiological study encompassing populations from 24 countries reported a PTSD prevalence of 17.52% [8]. Therefore, identifying early diagnostic biomarkers for PTSD is clinically imperative for timely detection and targeted intervention.

Metabolomics evaluates the overall metabolic status by systematically analyzing the dynamic changes in small-molecule metabolites within biological systems [9,10]. As direct products of cellular physiological activities, these metabolites are highly abundant and structurally diverse; moreover, their abundance is jointly influenced by endogenous physiological processes and exogenous environmental factors [11,12]. Therefore, the in-depth characterization of metabolite alterations and the metabolic pathways that they engage in represents a critical approach to uncovering the intrinsic mechanisms underlying the disease onset and progression [13]. In the field of PTSD research, multiple metabolomic studies have identified potential associations between specific metabolites and the disorder. Karabatsiakis et al. performed serum metabolomic profiling in 20 individuals with PTSD and 18 healthy controls, identifying palmitoylethanolamide and PE(17:1(9Z)/18:0) as the top candidates that were differentially expressed in the metabolites that were most strongly associated with PTSD [14]. Wei Y et al., through the deep mining of blood metabolite data from 7824 adults, found that higher circulating levels of palmitoyl sphingomyelin were associated with a reduced risk of developing PTSD [15,16]. Moreover, the global arginine bioavailability ratio (GABR) has been validated as a clinically relevant biomarker for PTSD. The plasma GABR was significantly reduced in 56 individuals with PTSD and exhibited significant negative correlations with inflammatory marker concentrations, PTSD symptom severity, negative affect, and adverse childhood experiences [17]. Tomoko Inoue et al. further focused on fatty acid profiles, analyzing 24 plasma fatty acid species at one and three months post-trauma in 39 trauma-exposed individuals; they found significant differences in plasma levels of arachidonic acid, palmitic acid, stearic acid, oleic acid, linoleic acid, α-linolenic acid, dihomo-γ-linolenic acid, and docosahexaenoic acid between those who developed PTSD and those who did not [18]. Collectively, these findings indicate that certain specific metabolites may be closely linked to the pathophysiological mechanisms of PTSD.

The microbiota–gut–brain axis (MGBA), a bidirectional communication network linking the gut microbiota, the gastrointestinal tract, and the central nervous system, has become a key focus in neuropsychiatric disease research. A growing body of evidence confirms that dysbiosis of the gut microbiota is closely associated with the onset and progression of multiple psychiatric disorders, including Alzheimer’s disease [19], Parkinson’s disease [20], major depressive disorder [21,22], schizophrenia [23], and PTSD [24]. With regard to PTSD specifically, numerous population-based and animal model studies have uncovered potential associations between alterations in the gut microbial composition and abnormalities in the brain function and behavior [25,26,27,28,29]. For example, Ruth Feldman et al. conducted a longitudinal cohort study of 148 individuals with long-term exposure to war-related environments and found that, during early childhood and adolescence, the relative abundance of the genus Dialister in the gut microbiota was significantly negatively correlated with PTSD symptom severity, whereas that of the genus Veillonella showed a significant positive correlation. More direct experimental evidence comes from fecal microbiota transplantation (FMT) studies: when fecal microbiota from adolescents diagnosed with PTSD were transplanted into germ-free mice, the recipient mice exhibited a marked increase in intestinal Clostridium ramosum abundance and concurrently displayed clear anxiety-like behaviors [30]. In another study of PTSD using a rat model, a 16S rRNA gene sequencing of gut microbiota revealed that the abundances of the phyla Firmicutes, Bacteroidetes, Cyanobacteria, and Proteobacteria were all significantly negatively correlated with serotonin levels in the brain [31]. Although it is widely acknowledged that the gut microbiota plays an important role in the pathophysiology of PTSD, the precise biological processes and underlying mechanisms remain poorly defined and require further in-depth investigation.

However, it remains unclear whether alterations in the gut microbiota in PTSD are systematically linked to changes in the host plasma metabolome and whether these two factors interact synergistically to influence disease progression. Therefore, in this study, a murine PTSD model was induced using the SPS&S paradigm to examine gut microbiota–plasma metabolite associations. The gut microbial and plasma metabolite profiles in PTSD mice were characterized via 16S rRNA gene sequencing and UHPLC–MS/MS. Furthermore, pathway enrichment analysis and correlation analysis were performed to explore the potential links between the altered microbial taxa and the metabolites.

## 2. Methods

### 2.1. Animals

C57BL/6 mice (aged 8 weeks, male, with body weights ranging from 18 to 22 g) were sourced from the Experimental Animal Center of the Army Medical University (Chongqing, China). The mice were housed in polypropylene cages under standardized environmental conditions: a 12 h light/12 h dark cycle, ambient temperature maintained at 20–22 °C, with ad libitum access to standard laboratory chow and water. Prior to experimentation, all mice underwent a 7-day acclimation period in the experimental facility. During the experiment, body weight changes in the mice were recorded (Supplementary Figure S1). All the animal experiments involved in this study were approved by the Ethics Committee of the Army Medical University (AMUWEC20225045), and all the animal treatments were performed under the National Institutes of Health guidelines.

### 2.2. PTSD Model Establishment

**Figure 1:**
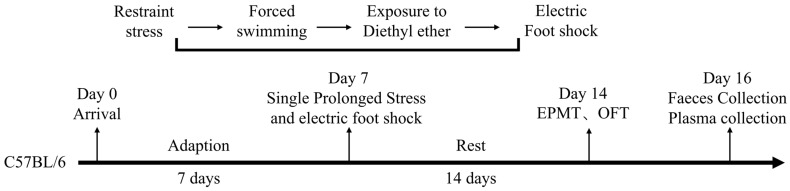
The experimental procedure for PTSD. EPMT, elevated plus maze test, and OFT, open field test.

Following the acclimation period, mice were randomly allocated to a control group and a PTSD model group (*n*  =  5/group). The PTSD model was subsequently established in the model group using the single prolonged stress and electric foot shock (SPS&S) paradigm (Figure 1). This protocol comprised four sequential stressors: restraint stress, forced swimming, general anesthesia, and an unconditioned foot shock stimulus. The detailed procedure was executed as follows: PTSD model group mice were confined in restraint devices for 4 h, then transferred to standard housing cages (50  ×  40  ×  30 cm) for 30 min of unrestricted movement. Subsequently, the mice were subjected to forced swimming in polypropylene containers (diameter 20 cm, height 30 cm) containing water (25 °C, depth 20 cm) for 20 min. Post-swimming, mice were returned to their housing cages for 30 min of recovery. The mice were then anesthetized via ether vapor inhalation until achieving surgical plane anesthesia, as evidenced by the absence of a toe pinch reflex and a depressed respiratory rate. After 30 min, the PTSD model mice were placed in an electrified grid chamber (27  ×  20  ×  30 cm) and administered a single unconditioned foot shock (0.8 mA, 5 s). The control group mice underwent identical chamber exposure for 5 s without electrical stimulation. All mice were finally returned to their housing cages under undisturbed conditions.

### 2.3. Behavioral Test

All behavioral tests were conducted by experienced experimenters blinded to group allocation. The testing occurred during the light phase of the diurnal cycle. At least 30 min prior to each test, mice with their home cages were transferred to the testing environment. Post-testing, all apparatus surfaces were sanitized with 75% ethanol solution and air-dried before their subsequent utilization.

### 2.4. Elevated Plus Maze Test

The mouse anxiety levels were assessed using the elevated plus maze test (EPMT). The maze comprised two open arms and two enclosed arms (arm dimensions: 28 cm length × 5.8 cm width), with the identical arm types positioned opposite each other. The enclosed arms were bounded by gray walls (height: 15.5 cm). The entire platform was elevated 55 cm above the floor, with arms converging at a central square (5 cm × 5 cm). During formal testing, mice were placed on the central platform facing an enclosed arm. The behavioral trajectories were continuously recorded for 5 min via a video tracking system. An arm entry was scored when the mouse’s trunk (including all limbs) completely crossed the arm entrance threshold. The parameters that were analyzed included the open arm duration and the open arm entry frequency.

### 2.5. Open Field Test

The open field test (OFT) was employed to quantify anxiety-like behavior in mice. The testing was conducted under low-illumination conditions in a sound-attenuated environment. The apparatus, constructed of black metal, measured 72 cm (L) × 72 cm (W) × 60 cm (H). The floor area was partitioned into 16 equidistant squares. During testing, each mouse was positioned in the central quadrant and permitted unrestricted exploration for 5 min. The behavioral parameters, including ambulatory crossings and rearing events, were digitally recorded via a video tracking system throughout the 5 min session.

### 2.6. Sample Collection and Preparation

Following anesthesia via intraperitoneal pentobarbital sodium administration, murine blood was collected through transcardial puncture. Anticoagulated whole blood was transferred to heparinized tubes, gently inverted for homogenization, and incubated at room temperature for 30 min. The centrifugation protocols were implemented as follows: primary centrifugation at 3000× *g* for 10 min with supernatant harvest, secondary centrifugation of supernatant at 5000× *g* for 3 min, and the resultant supernatant was aliquoted and cryopreserved at −80 °C. Concurrently, aseptic surgical dissection was performed to excise intestinal tracts. The fresh fecal specimens were collected per mouse in 2 mL sterile microcentrifuge tubes and similarly cryopreserved at −80 °C for subsequent microbiomic DNA extraction and 16S rRNA gene sequencing. All the animals were sacrificed, and the samples were collected between 9:00 and 11:00 a.m.

### 2.7. Cytokine Analysis

The plasma samples were retrieved from the −80 °C freezer and equilibrated to room temperature. The levels of IL-1β and TNF-α in serum were measured using enzyme-linked immunosorbent assay (ELISA). The assay was performed using Elabscience High Sensitivity kits (Elabscience, Wuhan, China), with detection ranges of 3.13–200 pg/mL for IL-1β and 4.69–300 pg/mL for TNF-α. The standards and experimental serum samples (100 μL) were added to 96-well plates in triplicate and incubated at 37 °C for 90 min. The liquid was then removed, and 100 μL of the biotinylated antibody working solution was added to each well, followed by incubation at 37 °C for 1 h. The wells were then washed three times with wash buffer. Next, 100 μL of HRP-conjugate working solution was added to each well and incubated at 37 °C for 30 min, followed by five washes. Subsequently, 90 μL of substrate solution (TMB) was added to each well and incubated at 37 °C for 15 min in the dark. The optical density was immediately measured at 450 nm using a microplate reader. The intra- and inter-assay coefficients of variation for all the analyses were below 10%.

### 2.8. Metabolomics Analysis

Following thawing on ice at 4 °C, plasma aliquots (100 μL) were immediately combined with 400 μL of pre-chilled methanol/acetonitrile (1:1, *v*/*v*). The mixture was vortex-mixed (30 s) and subjected to an ice-water bath sonication (5 min). The protein precipitation was achieved by incubating at −20 °C (1 h), followed by centrifugation (13,000× *g*, 15 min, 4 °C). The supernatant was lyophilized in a vacuum concentrator. For UHPLC-MS/MS analysis, the lyophilizate was reconstituted in 100 μL reconstitution solvent [acetonitrile/water (1:1, *v*/*v*)], re-centrifuged (13,000× *g*, 15 min, 4 °C), and the resultant supernatant was injected for analysis. The quality control (QC) samples were generated by pooling equal volumes (15 μL) of individual supernatants, with interleaved QC injections and monitoring the instrumental signal stability throughout the sequence.

The LC-30A UHPLC system (Shimadzu, Kyoto, Japan) was operated in tandem with a TripleTOF 4600 system (SCIEX, Framingham, MA, USA). The separation steps were conducted by hydrophilic interaction liquid chromatography (HILIC) separation and reversed-phase liquid chromatography (RPLC) separation. A Kinetex C18 column (2.1 mm × 100 mm, 2.6 μm, 100 Å, Phenomenex, Torrance, CA, USA) was used for the binary gradient elution. Solvent A consisted of 0.1% formic acid in water (*v*/*v*), and solvent B comprised 0.1% formic acid in acetonitrile (*v*/*v*). The flow rate was 0.35 mL/min with an injection volume of 2 μL. The gradual increase program was a gradient from 15% B (0 min) to 85% B (10 min), with a total of 15 min run time. The TSKgel NH2-100 column (2.1 mm × 100 mm, 3.0 μm, Tosoh, Tokyo, Japan) was used, with a binary elution gradient program. Solvent A was a 5 mM solution of ammonium acetate, and solvent B was acetonitrile. The column flow rate was a flow rate of 0.25 mL/min, and the injection volume was 2 μL. The gradient started from 100% B in 2 min, then switched to 15% B at 15 min, followed by 100% B at 20 min, with a total run time of 25 min.

### 2.9. Metabolomic Data Processing and Analysis

The raw data were converted to a mzXML format using ProteoWizard MSConvert, followed by peak alignment, retention time correction, and peak area extraction with XCMS software (Bioconductor, Boston, MA, USA). The peak picking was performed with the following parameters: centWave *m*/*z* = 10 ppm, peakwidth = c(10, 60), and prefilter = c(10, 100). For peak grouping, bw = 5, mzwid = 0.025, and minfrac = 0.5 were applied. CAMERA was used for the annotation of isotopes and adducts. Among the extracted ion features, only variables with more than 50% nonzero measurement values in at least one group were retained. The metabolite identification was achieved by comparing accurate *m*/*z* values (<10 ppm) and MS/MS spectra with an in-house database established using available authentic standards. The confidence level for metabolite identification was level 2 or higher.

The preprocessed data were loaded into the R software environment. First, unsupervised principal component analysis (PCA) was performed to detect and exclude the potential outlier data points. Subsequently, the partial least squares discriminant analysis (PLS-DA) and orthogonal partial least squares discriminant analysis (OPLS-DA) were carried out. To evaluate the reliability and stability of the model, 200 permutation tests were conducted on each discriminant analysis model, and overfitting was assessed by comparing the intercept values of R^2^ and Q^2^ in the permutation test results. Concurrently, univariate statistical analysis was performed to screen metabolites that met both conditions of *p* < 0.05 and VIP ≥ 1, which were designated as significantly different metabolites. The screened differential metabolites were annotated utilizing the Human Metabolome Database (HMDB) (http://www.hmdb.ca/; accessed on 15 October 2025). Finally, the identified significantly differentially expressed metabolites were imported into the Kyoto Encyclopedia of Genes and Genomes (KEGG) (http://www.genome.jp/kegg/; accessed on 15 October 2025), and the pathway enrichment analysis was executed on the database to construct the metabolic pathways associated with these differential metabolites.

### 2.10. 16S rRNA Sequencing of Gut Microbiota

The total genomic DNA was extracted from fecal samples using the OMEGA Soil DNA Kit (Omega Bio-Tek, Norcross, GA, USA). The concentration and purity of the extracted DNA were assessed through 1% agarose gel electrophoresis and ultraviolet spectrophotometry (measuring the A260/A280 ratio). Based on concentration measurements, an appropriate volume of DNA was aliquoted for subsequent analysis. PCR amplification of the V3-V4 hypervariable region of the bacterial 16S rRNA gene was conducted with primers 515F (5′-GTGYCAGCMGCCGCGGTAA-3′) and 806R (5′-GGACTACNVGGGTWTCTAAT-3′). The thermocycling protocol comprised initial denaturation at 94 °C for 1 min (1 cycle); followed by 30 cycles of denaturation at 94 °C for 20 s, annealing at 54 °C for 30 s, and extension at 72 °C for 30 s; concluding with a final extension at 72 °C for 5 min (1 cycle); and finally storing the product at 4 °C. The target amplicon (~400–450 bp) was purified using the QIAquick Gel Extraction Kit (Qiagen, Hilden, Germany). The purified products were subjected to quantification via the QuantiFluor™ ST Fluorescence System (Promega, Madison, WI, USA). The qualified libraries were ultimately sequenced using paired-end 250 bp (PE250) chemistry on the Illumina NovaSeq platform (Illumina, San Diego, CA, USA).

### 2.11. Bioinformatic and Statistical Analysis of 16S rRNA Data

The original FASTQ file was processed with the QIIME 2 package software (v2020.2; https://qiime2.org; Qiime 2 Development Team, Boulder, CO, USA) for data quality control, and an operational taxonomic unit (OTU) table was generated based on the filtered sequence data. After that, alpha diversity indicators, including the Chao1, Shannon and Simpson indices, were calculated to indicate the level of species richness and diversity of the microbial community. For comparison of the difference in bacterial community structure in the control group and PTSD group (n = 5 for each group), β diversity indexes were determined and visualized by principal coordinates analysis (PCoA) to identify the overall differences and similarities of bacterial communities across the sample groups. We also used the linear discriminant analysis effect size (LEfSe) method to screen the microbial communities with significant intersample differences, which could be candidate biomarkers.

### 2.12. Spearman Correlation Analysis

To assess the correlations between the specific metabolites and the gut microbiota at the OTU level, Spearman’s rank correlation coefficient was employed. Prior to analysis, the metabolite concentration data and microbial OTU counts were transformed using log(x + 1) to reduce the data skewness and stabilize variance, which is a standard approach for handling compositional datasets. All statistical analyses were conducted in R (version 4.4.3) using the corr.test function from the psych package, with the argument method set to “spearman”. To control for the false discovery rate associated with multiple comparisons, the resulting *p*-values were adjusted using the Benjamini–Hochberg procedure, with an adjusted *p* < 0.05 considered statistically significant.

### 2.13. Statistical Analysis

All the statistical analyses were performed using SPSS 20.0 software (SPSS Inc., Chicago, IL, USA). The normality was confirmed for all variables through empirical testing. The one-way analysis of variance (ANOVA) was employed to identify significantly dysregulated molecules. Independent samples Student’s *t*-tests and the Benjamini–Hochberg procedure were carried out to compare intergroup differences and screen differentially expressed metabolites and microorganisms. The statistical significance thresholds were designated as follows: *p* < 0.05 indicated statistical significance, and *p* < 0.01 denoted high statistical significance.

## 3. Results

### 3.1. Elevated Plus Maze Test

**Figure 2:**
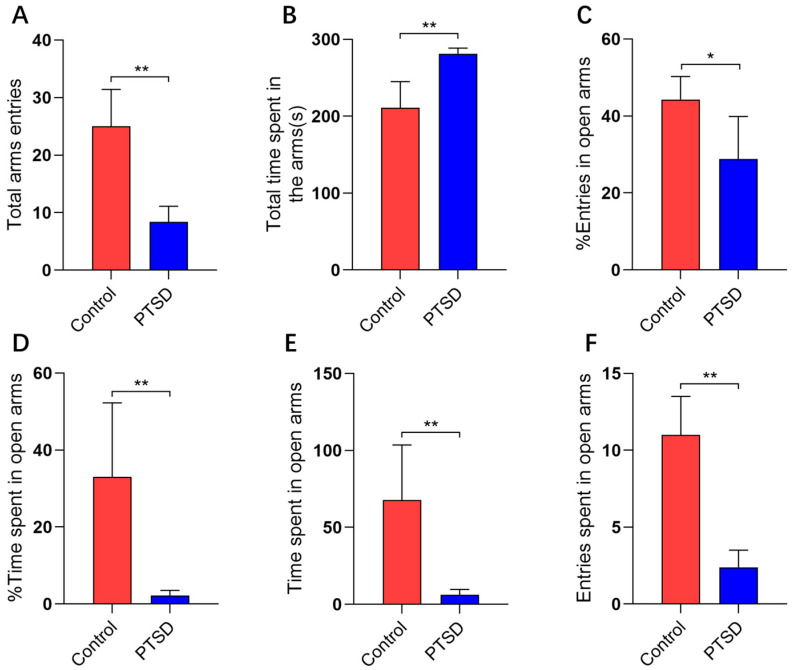
The evaluation of anxiety levels in mice induced by SPS&S using the elevated plus maze test (n = 5). (**A**) The total arm entries; (**B**) the total arm dwell time; (**C**) the percentage of open arm entries; (**D**) the percentage of open arm entry time; (**E**) the open arm dwell time; and (**F**) the open arm average entries. The data are presented as mean ± standard deviation (mean ± SD). * *p* < 0.05, and ** *p* < 0.01.

The elevated cross maze test was employed to assess the anxiety levels of mice subjected to SPS&S. Significant differences were observed between the control and PTSD groups regarding total arm entry frequency (Figure 2A) and total arm dwell time (Figure 2B). The SPS&S induction markedly decreased the percentage of open arm access times, with values of 42.3% for the control group and 32.4% for the PTSD group (Figure 2C). The SPS&S induction also significantly reduced the percentage of open arm entry time, with 29.4% for the control group and 2.2% for the PTSD group (Figure 2D). The observed dwell time in the open arm was 365 s for the control group and 38 s for the PTSD group (Figure 2E). The number of open arm entries was 62 for the control group and 14 for the PTSD group (Figure 2F).

### 3.2. Open Field Test

**Figure 3:**
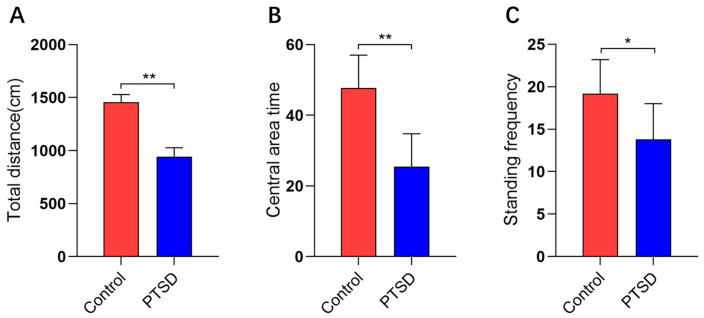
The open field test assessed anxiety levels in mice subjected to SPS&S (*n* = 5). (**A**) The total distance traveled, (**B**) the central area time, and (**C**) the standing frequency. The data are presented as mean ± standard deviation (mean ± SD). * *p* < 0.05, and ** *p* < 0.01.

The anxiety levels of mice induced by SPS&S were evaluated utilizing open field experiments. The SPS&S induction significantly reduced the total locomotion distance, with values of 1458.0 cm in the control group versus 943.5 cm in the PTSD group (Figure 3A). Furthermore, the SPS&S induction markedly decreased the central zone duration and rearing frequency. The central zone duration measured 47.8 s for the controls and 25.5 s for the PTSD group (Figure 3B). The standing frequency was recorded at 19.2 episodes in the controls and 13.8 episodes in the PTSD group (Figure 3C).

### 3.3. Cytokines

**Figure 4:**
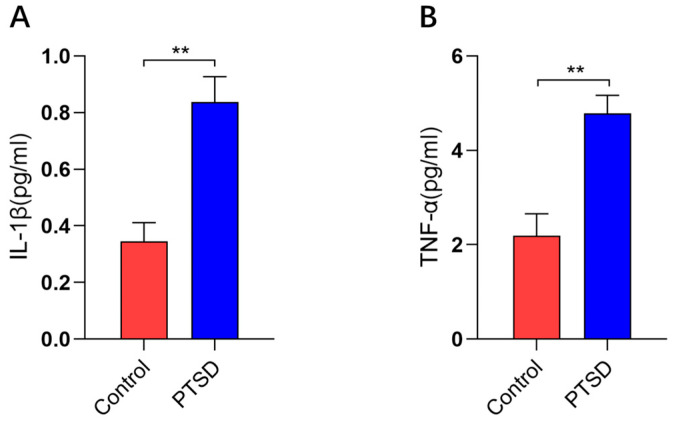
The plasma cytokine concentrations in control and PTSD mice (*n* = 5). (**A**) ELISA was used to quantify IL-1β, and (**B**) ELISA was used to quantify TNF-α. The data are presented as mean ± standard deviation (mean ± SD). ** *p* < 0.01.

Following behavioral testing, the plasma inflammatory factor levels were quantified in the PTSD murine model. The plasma concentrations of Interleukin-1β (IL-1β, *p* < 0.01) and Tumor Necrosis Factor-α (TNF-α, *p* < 0.01) were significantly elevated in PTSD model mice compared with the controls (Figure 4A,B).

### 3.4. Metabolomics Analysis

**Figure 5:**
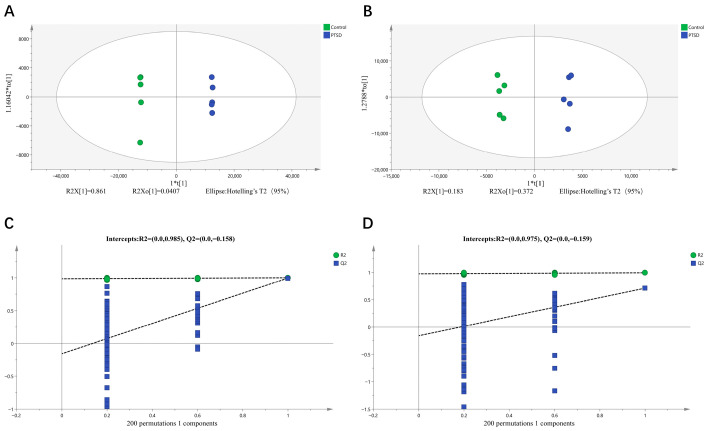
The OPLS-DA score plots and the OPLS-DA quality control chart of mouse plasma samples. (**A**) The OPLS-DA score plot for positive ion mode; (**B**) the negative ion mode OPLS-DA score plot; (**C**) the positive ion mode OPLS-DA replacement test; and (**D**) the negative ion mode OPLS-DA replacement test. The dashed lines indicate the intercepts of R^2^ and Q^2^ from 200 permutation tests. Q^2^ intercept < 0.05 indicates no overfitting. “*” Representing separators.

UHPLC-MS/MS was used to determine the plasma metabolic profiles in the control mice and the PTSD mouse model. The QC data had excellent experimental stability, reproducibility and reliability of data (Supplementary Figure S2). The PCA model was identified in all the metabolites of the two clusters, separating the controls and the PTSD participants, confirming the difference in the metabolic features (Supplementary Figure S3). Furthermore, OPLS-DA made a clear grouping difference between the groups in positive ion mode (R2X = 0.951, R2Y = 1.0, Q2 = 0.996; Figure 5A) and negative ion mode (R2X = 0.766, R2Y = 0.994, Q2 = 0.711; Figure 5B), thus confirming the substantial changes in PTSD plasma metabolomes. We performed two hundred permutation tests for positive ion mode (R2 = 0.985, Q2 = −0.158; Figure 5C) and negative ion mode (R2 = 0.975, Q2 = −0.159; Figure 5D), which did not overfit, thus verifying the reliability and predictability of the OPLS-DA model.

### 3.5. Differential Metabolites

**Figure 6:**
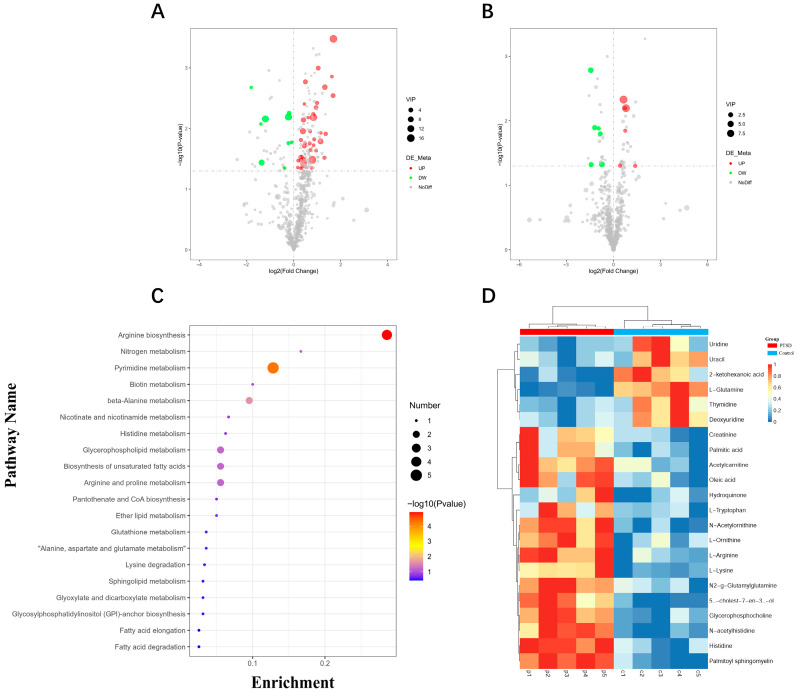
The plasma metabolite profile alterations in control and PTSD model mice. (**A**) The volcano plot depicting differential metabolites in positive ion mode; (**B**) the volcano plot depicting differential metabolites in negative ion mode; (**C**) the KEGG pathway enrichment analysis of differentially expressed metabolites; and (**D**) the heatmap of the differentially expressed metabolites. The vertical dashed line represents the fold change threshold (|log_2_FC| ≥ 0.5), and the horizontal dashed line represents the statistical significance threshold (−log_10_P ≥ 1.3, P < 0.05).

Utilizing non-targeted metabolomics, 1177 plasma metabolites were profiled in control and PTSD murine cohorts. Based on screening criteria (VIP > 1, *p* < 0.05), 24 significantly differential plasma metabolites were identified (Figure 6A,B, Table 1). The cluster analysis demonstrated distinct separation patterns between the groups (Figure 6D). The KEGG pathway enrichment analysis revealed predominant involvement in: arginine biosynthesis, pyrimidine metabolism, unsaturated fatty acid biosynthesis, glycerophospholipid metabolism, arginine and proline metabolism, nicotinate and nicotinamide metabolism, and lysine degradation (Figure 6C).

### 3.6. Gut Microbial Community Diversity Analysis

**Figure 7:**
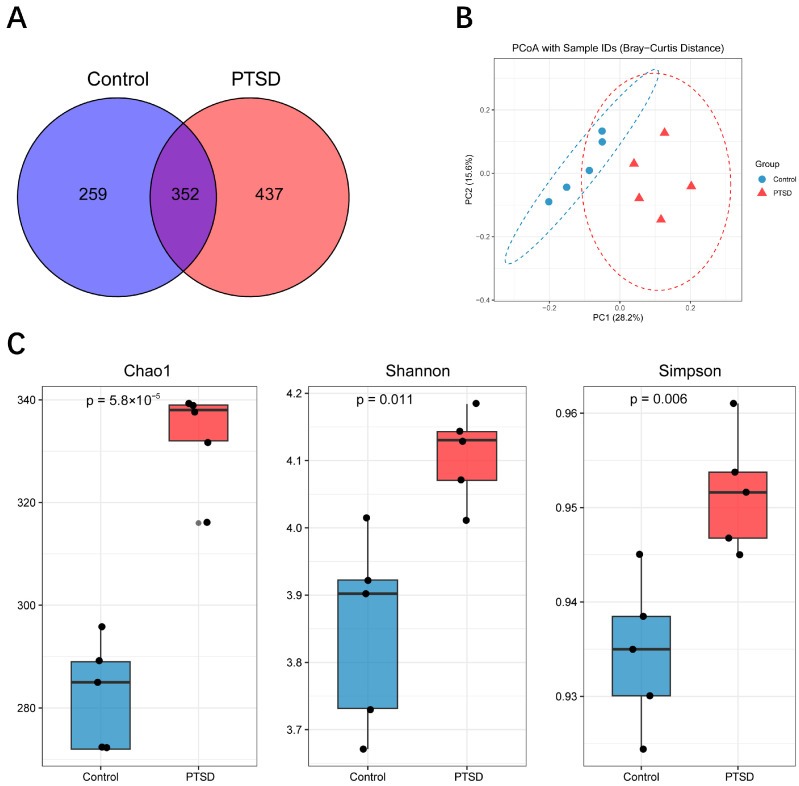
The gut microbiota characteristics of the control group and the PTSD group. (**A**) The Venn diagram of OTU distribution between the control and PTSD group samples; (**B**) at the OTU level, PCoA demonstrated a significant difference (*n* = 5) in gut microbiota composition between the control group and the PTSD group; and (**C**) the Chao1, Shannon, and Simpson diversity indices of the gut microbiota in the control group and the PTSD group.

**Figure 8:**
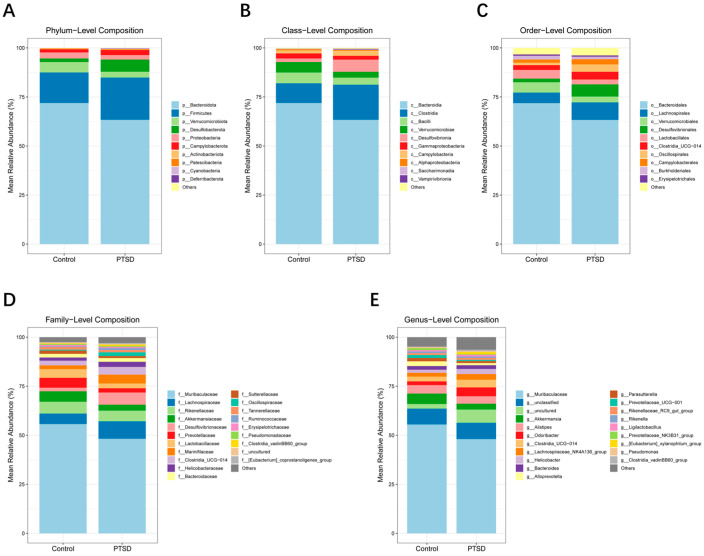
The abundance of gut microbiota at the phylum to genus level in the control group and the PTSD group. (**A**) The abundance of gut microbiota at the phylum level; (**B**) the abundance of gut microbiota at the class level; (**C**) the abundance of gut microbiota at the order level; (**D**) the abundance of gut microbiota at the family level; and (**E**) the abundance of gut microbiota at the genus level.

The gut microbiota was detected by 16S rRNA sequencing, with an average of 44,000 high-quality clean reads per sample. The cluster analysis identified 1048 OTUs. Among them, there were 259 OTUs exclusive to the control group and 437 OTUs unique to the PTSD group (Figure 7A). The richness and diversity of microbial communities in the two groups were analyzed by alpha diversity. The CHAO1 index, Shannon index and Simpson index all showed significantly higher indices in the PTSD group than the control group (*p* < 0.05), and it has been suggested that PTSD may alter the richness and diversity of the gut microbiota (Figure 7B). By analyzing the beta diversity through PCoA, PCoA showed that there was a significant separation of the control group and the PTSD group (Figure 7C). Meanwhile, the OTUs were annotated, and then the microorganisms were analyzed at different taxonomic levels. At the level of phylum and class, the most dominant phylum and class were the phylum Bacteroidota and class Bacteroidia, respectively, and they represented >60% (Figure 8A,B). At the order level, Bacteroidales, Lachnospirales, and Verrucomicrobiales dominated over 75% of the orders that were detected in the gut (Figure 8C). At the family level, the dominant taxa were Muribaculaceae, Lachnospiraceae, and Akkermansiaceae (Figure 8D). At the genus level, the highest abundance of the gut microbiota was found in the Muribaculaceae family (Figure 8E). There was a significant difference in the relative abundance of the gut microbiota at the genus level between the control and PTSD groups.

### 3.7. LEfSe Analysis

**Figure 9:**
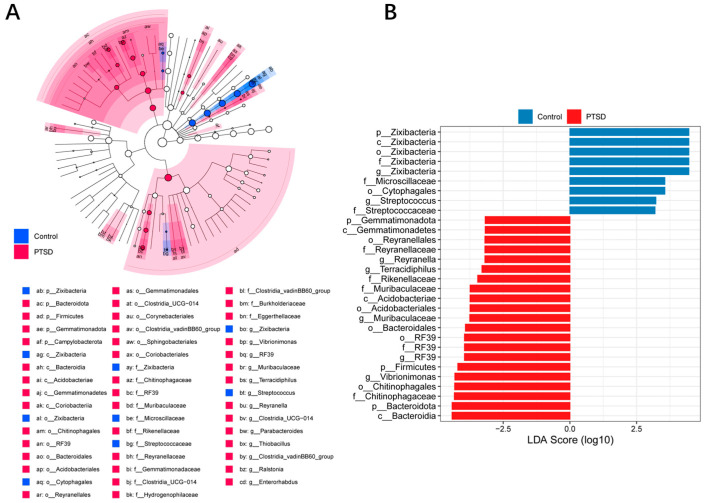
The relative abundance of gut microorganisms in the control group and the PTSD group. (**A**) A cladogram of the differentially abundant bacteria of the two groups. (**B**) A histogram of the LDA score for the differential abundance of the bacteria between the two groups. (LDA score > 2.0, *p* < 0.05).

The LEfSe method was used to compare the characteristics of gut bacteria between the control group and the PTSD group. The differences were ascertained with an LDA score > 2.0, *p* < 0.05. As shown in Figure 9A,B, specific bacterial groups of the control group at the genus level were Zixibacterium and Streptococcus, respectively. The signature groups of the PTSD group at the genus level were Reyranella, Terracidiphilus, Muribaculaceae and Vibrioimonas.

### 3.8. Correlation of Gut Microbiota with Plasma Metabolites

**Figure 10:**
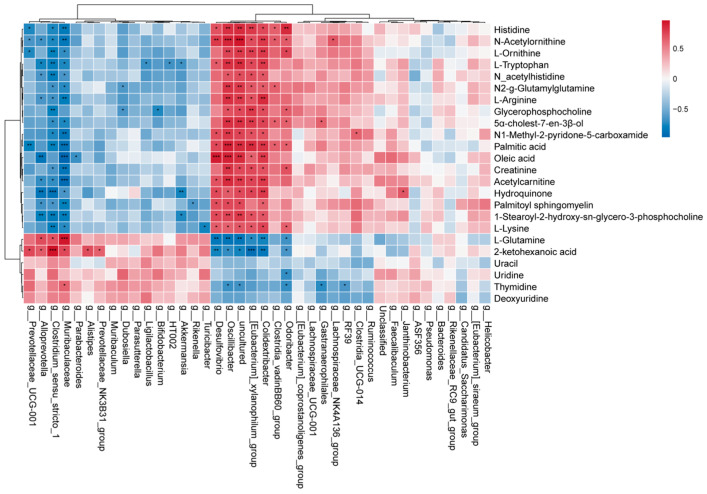
Spearman correlation heatmap of metabolites and gut microbiota at the genus level. * *p* < 0.05, ** *p* < 0.01, and *** *p* < 0.001.

For insights into the functional link between gut microbiota and the levels of plasma metabolites in the mouse PTSD model, Spearman’s correlation analysis was performed at the genus level (Figure 10). Significant associations were observed: correlations of members of Muribaculaceae with L-glutamine (*p* < 0.001), 2-ketohexanoic acid (*p* < 0.05) and thymidine (*p* < 0.05) were positive, but the associations with palmitic acid (*p* < 0.001), oleic acid (*p* < 0.001), L-arginine (*p* < 0.01) and palmitoyl sphingomyelin (*p* < 0.01), and L-ornithine (*p* < 0.05) were negative; Akkermansia was negatively correlated with Hydroquinone (*p* < 0.01) and L-tryptophan (*p* < 0.05). The Lachnospiraceae_NK4A136_group had a positive correlation with N-Acetylornithine (*p* < 0.05). These observations indicate functional interplay between intestinal microbiota and circulating metabolites.

## 4. Discussion

Post-traumatic stress disorder exhibits complex etiological mechanisms and is frequently comorbid with emotional dysregulation, such as anxiety. The clinical translation of biomarkers and therapeutic targets remains limited, with molecular pathogenesis yet to be fully elucidated. This study employed SPS&S combined with plantar electrical stimulation to simulate traumatic conditions [32], successfully establishing a PTSD murine model validated through behavioral assessments, including the EPMT and OFT. The results demonstrated that compared to controls, the PTSD model mice exhibited significant reductions in the open-arm entry percentage (*p* < 0.05) and the time spent in open arms during EPMT (*p* < 0.05), alongside decreased total locomotion distance (*p* < 0.05) and grid crossings in OFT (*p* < 0.05), indicating hypoactivity and diminished exploratory behavior. Furthermore, plasma levels of pro-inflammatory cytokines TNF-α (*p* < 0.01) and IL-1β (*p* < 0.05) were significantly elevated in the PTSD group, aligning with the established literature [33,34,35]. The metabolomic profiling revealed disruptions in the plasma metabolites, including L-arginine, ornithine, palmitic acid, oleic acid, uridine, creatinine, histidine, L-tryptophan, L-lysine, and uracil. Differential metabolites were significantly enriched in arginine biosynthesis, pyrimidine metabolism, unsaturated fatty acid biosynthesis, glycerophospholipid metabolism, arginine and proline metabolism, niacin/nicotinamide metabolism, and lysine degradation pathways. Concurrently, gut microbiota dysbiosis was observed in the PTSD group, characterized by the significantly reduced abundance of beneficial taxa, including Muribaculaceae and Akkermansia.

We observed significantly elevated levels of L-arginine and its metabolite L-ornithine in the plasma of the PTSD mouse model, alongside increased circulating levels of the inflammatory cytokines TNF-α and IL-1β. As a multifunctional semi-essential amino acid, arginine can be metabolized via the arginase pathway into ornithine and polyamines, the latter playing a key role in tissue repair and the regulation of late-stage inflammatory responses [36,37,38,39]. In a GABR study, PTSD subjects exhibited significantly higher serum ornithine and significantly lower arginine compared to the controls [17]. Of note, patients with bipolar disorder (BD) show significantly elevated serum arginine relative to healthy controls [40]. The concurrent increase in L-arginine and ornithine observed in our study suggests this may represent a systemic metabolic adaptation to traumatic stress. Therefore, we speculate that a sustained peripheral inflammatory state may drive the activation of the arginine metabolism, while the competitive activation of the arginase pathway promotes the accelerated accumulation of ornithine. Therefore, in future studies, we will measure arginase activity to validate the hypothesis of its relative activation in the context of PTSD. Concurrently, dynamic monitoring of the post-traumatic trajectories of plasma arginine and ornithine will be performed to further elucidate the relationship between the L-arginine-ornithine axis and PTSD.

This study found that plasma levels of palmitic acid (PA) were significantly elevated in the SPS&S-induced PTSD model mice, and showed a significant positive correlation with TNF-α and IL-1β levels, accompanied by a concurrent increase in oleic acid (OA) levels and an increase in palmitoyl sphingomyelin levels. This finding is highly consistent with recent clinical observations in PTSD patients [41]. A study by Ogawa et al. involving 68 patients with post-traumatic stress disorder and 97 healthy controls revealed that plasma saturated palmitic acid levels were significantly higher in the PTSD patients compared to the healthy controls, and were significantly positively correlated with inflammation-related markers, whereas oleic acid levels were associated with inflammatory markers. Notably, the positive correlation between PA and TNF-α and IL-1β observed in the animal model in this study aligns with the association pattern between PA and inflammatory markers reported in the aforementioned clinical study, suggesting that the pathophysiological processes underlying PTSD may be conserved across species. However, elucidating the specific roles of PA and OA in the pathophysiology of PTSD and their causal relationship with inflammation will require further functional experiments and clinical studies. The increase in palmitoyl sphingomyelin levels observed in this study currently lacks direct evidence from studies within the field of PTSD. Sphingomyelin is an essential component of cell membranes and a precursor for signaling molecules such as ceramides; its metabolic alterations may be involved in the regulation of inflammatory signals [42,43,44], but the biological significance of this change awaits further experimental validation.

This study confirms that there were significantly decreased plasma concentrations of uridine and uracil in the PTSD mouse model, which is consistent with our previous report of reduced urinary uridine [45]. Uridine, a pyrimidine metabolite with neuroprotective properties, can be transported via the circulation to the cerebrospinal fluid and brain extracellular space [46,47], where it exerts anxiolytic and anticonvulsant effects [48]. A deficiency in uridine may compromise the central nervous system’s protective mechanisms, rendering the brain more susceptible to peripheral inflammatory factors [49,50]. The study observed a potential association between reduced uridine levels and elevated inflammatory cytokines. However, whether the reduction in uridine is directly mediated by a central protective mechanism or represents a systemic response to traumatic stress cannot be inferred directly from the association analysis, providing important clues for future investigations into the mechanisms underlying PTSD.

The metabolic pathway enrichment analysis revealed that the differential metabolites were primarily enriched in pathways such as arginine biosynthesis, unsaturated fatty acid biosynthesis, and pyrimidine metabolism, suggesting that traumatic stress can induce global perturbation of these metabolic networks. Among these, palmitic acid, L-arginine, and ornithine exhibited covarying trends with peripheral inflammatory cytokines, whereas the reduction in uridine levels may be involved in central protective mechanisms. However, whether these metabolic alterations represent a direct consequence of traumatic stress or manifestations of adaptive regulation cannot be inferred from the association analysis, and subsequent studies will integrate molecular functional assays and intervention experiments to further elucidate the specific roles of these metabolic pathways in the pathological progression of PTSD.

The phyla Firmicutes and Bacteroidetes serve as core components of the human gut microbiota, playing a crucial role in maintaining normal gastrointestinal function [51]. Based on 16S rRNA sequencing analysis at the phylum level, we observed that Firmicutes and Bacteroidetes exhibited the highest abundance. Furthermore, the Bacteroidetes/Firmicutes ratio showed a significant difference between the two groups. At the family level, the PTSD model group displayed characteristic dysbiosis: significantly decreased abundance of Muribaculaceae, Akkermansiaceae, Prevotellaceae, and Lactobacillaceae, while Lachnospiraceae and Desulfovibrionaceae showed a significantly increased abundance. These results indicate that PTSD induces a profound dysregulation of murine gut microbiota.

To further explore the mechanism of action of the gut microbiota in the PTSD model mice, the analysis at the genus level revealed a significant decrease in the abundances of the Muribaculaceae and Akkermansia, while the abundance of the Lachnospiraceae NK4A136_group increased significantly. Among them, Muribaculaceae is the dominant anaerobic taxon in the mouse intestine [52], possessing antioxidant properties and stress resilience [53]. An increase in its abundance improves the intestinal microenvironment homeostasis, promotes the synthesis of beneficial metabolites, and ameliorates anxiety-like behaviors [54]. The decrease in Akkermansia abundance represents a hallmark feature of low-grade inflammatory conditions in both humans and mice [55,56]; the increase in the abundance of Lachnospiraceae NK4A136_group demonstrates established associations with anxiety, depression, cognitive impairment [57], and vestibular dysfunction accompanied by neuropathic pain [58]. It is worth noting that the LEfSe analysis suggests that Muribaculaceae may serve as a potential PTSD biomarker or participate in pathological processes through the synergistic effects of microbiota.

In this study, the integrated analysis of gut microbiota and plasma metabolome revealed that Muribaculaceae was significantly decreased in the PTSD model mice and showed a significant negative correlation with PA and OA. Meanwhile, PA and OA were positively correlated with the levels of inflammatory cytokines, suggesting that both may be involved in the regulation of inflammatory processes. Combined with the reduction in plasma uridine and elevated inflammatory cytokines, the disturbances in pyrimidine metabolism, arginine biosynthesis, and unsaturated fatty acid metabolism indicated by metabolic pathway enrichment analysis, as well as the gut microbiota dysbiosis, further suggest the existence of potential interactions among microbiota, metabolites, and inflammatory status during the pathological process of PTSD.

## 5. Limitations

Although our study identified distinct gut microbiota and plasma metabolites in the PTSD mouse model, several limitations should be noted. First, the small sample size (*n* = 5 per group) limits statistical power; thus, the correlation findings should be considered exploratory and require validation in larger cohorts. Second, while we observed systemic inflammation in the PTSD model, we did not assess inflammatory cytokine expression or neuronal status in the brain tissue, precluding direct confirmation of the mechanisms by which systemic inflammation affects the nervous system. Third, we did not measure the food/water intake, the fecal output, the intestinal transit time, or the intestinal permeability in the mice, leaving discussions of altered gut function speculative. Fourth, the cross-sectional design precludes causal inferences regarding the associations among gut microbiota, plasma metabolites, and behavioral phenotypes. Future studies should incorporate metabolic cage monitoring, and functional experiments such as fecal microbiota transplantation, and intervention studies are warranted to establish the causal relationships between the phenotypes and the underlying mechanisms.

## 6. Conclusions

This study examined the interplay between the gut microbiota and the plasma metabolites in mice modeling PTSD, using 16S rRNA gene sequencing coupled with UHPLC-MS/MS. The gut microbiome data showed that PTSD caused highly disordered gut microbiota in mice, and the Muribaculaceae strain that was screened may be a potential biomarker for PTSD model mice. The plasma metabolomic profiling identified arginine biosynthesis, unsaturated fatty acid biosynthesis, and pyrimidine metabolism as the top pathways enriched among differentially abundant metabolites between the PTSD and control mice. The Spearman correlation analysis uncovered robust associations between specific microbial taxa and plasma metabolite abundances. These results suggest that gut microbiota and plasma metabolites also contribute to the pathophysiology of PTSD and provide a basis for future mechanistic investigation.

## Figures and Tables

**Table 1:** The significantly differentiated metabolites in the control group and the PTSD group.

No	Metabolites	FC	*p*-Value	Adj. *p*-Value	VIP	Trend
1	1-Stearoyl-2-hydroxy-sn-glycero-3-phosphocholine	5.18128	1.31926 × 10^−8^	1.72531 × 10^−6^	7.04406	↑
2	L-Glutamine	0.14185	6.57121 × 10^−7^	2.57811 × 10^−5^	2.32125	↓
3	Palmitoyl sphingomyelin	1.86521	1.88291 × 10^−6^	5.27662 × 10^−5^	5.016	↑
4	N-acetylhistidine	2.45764	1.29928 × 10^−5^	2.31705 × 10^−4^	1.22055	↑
5	Histidine	4.01037	1.35614 × 10^−5^	2.28026 × 10^−4^	2.5638	↑
6	N-Acetylornithine	2.49663	2.26593 × 10^−5^	3.41922 × 10^−4^	1.90844	↑
7	Glycerophosphocholine	2.09156	2.48749 × 10^−5^	3.61454 × 10^−4^	7.36942	↑
8	5α-cholest-7-en-3β-ol	4.03121	6.21691 × 10^−5^	6.41869 × 10^−4^	1.71005	↑
9	N2-g-Glutamylglutamine	3.52854	7.68233 × 10^−5^	7.47282 × 10^−4^	1.83453	↑
10	L-Lysine	2.14282	9.18155 × 10^−5^	8.57673 × 10^−4^	2.14269	↑
11	L-Arginine	1.82076	0.00011	0.001	2.41993	↑
12	L-Ornithine	1.93682	0.00041	0.00269	1.0943	↑
13	N1-Methyl-2-pyridone-5-carboxamide	2.29938	0.00053	0.00329	1.45207	↑
14	Acetylcarnitine	2.04835	0.00078	0.00436	2.17828	↑
15	2-ketohexanoic acid	0.36609	0.00164	0.00817	3.56613	↓
16	Palmitic acid	1.55761	0.00466	0.02004	9.30956	↑
17	Creatinine	1.56381	0.00618	0.02534	1.09553	↑
18	Oleic acid	1.73006	0.00634	0.02564	9.0672	↑
19	Thymidine	0.43574	0.01278	0.04657	2.98816	↓
20	Deoxyuridine	0.51843	0.01316	0.04764	1.45515	↓
21	L-Tryptophan	2.15465	0.01503	0.05311	1.18498	↑
22	Uracil	0.53818	0.02080	0.06976	0.98912	↓
23	Uridine	0.37175	0.04768	0.13621	2.57269	↓
24	Hydroquinone	2.60759	0.04993	0.14025	1.2828	↑

## Data Availability

The raw 16S rRNA gene sequencing data generated in this study have been deposited in the NCBI Sequence Read Archive (SRA) under the BioProject accession number PRJNA1418332.

## Supplementary Materials

The following supporting information can be downloaded at https://www.mdpi.com/article/10.3390/metabo16040222/s1. Figure S1. Body weight changes of the experimental mice were recorded (*n* = 5). Figure S2: The Pearson’s correlation coefficient among QC samples; Figure S3: PCA Results. Table S1: Fold changes (FC) and Variable importance in projection (VIP) score for metabolomics datasets for the PTSD and Control groups.

## Abbreviations

The following abbreviations are used in this manuscript:

PTSD	Post-traumatic stress disorder
SPS&S	Single prolonged stress and electric foot shock
UHPLC-MS/MS	Ultra-high-performance liquid chromatography–tandem mass spectrometry
GABR	Global arginine bioavailability ratio
MGBA	Microbiota–gut–brain axis
FMT	Fecal microbiota transplantation
EPMT	Elevated plus maze test
OFT	Open field test
PCA	Principal component analysis
PLS-DA	Partial least squares discriminant analysis
OPLS-DA	Orthogonal partial least squares discriminant analysis
HMDB	Human metabolome database
KEGG	Kyoto encyclopedia of genes and genomes
OUT	Operational taxonomic units
PCoA	Principal coordinates analysis
LEfSe	Linear discriminant analysis effect size
ANOVA	Analysis of variance
IL-1β	Interleukin-1β
TNF-α	Tumor necrosis factor-α
BD	Bipolar disorder
PA	Palmitic acid
OA	Oleic acid
AMPK	AMP-activated protein kinase
