# Supplementary material for: Correlation Between Gut Microbiota and Plasma Metabolites in a Mouse Model for Post-Traumatic Stress Disorder
Source: Metabolites. 2026 Mar 28;16(4):222. doi: 10.3390/metabo16040222 (PMC13118251; doi:10.3390/metabo16040222)
Supplement: Supplementary file 1 [file metabolites-16-00222-s001.zip › metabolites-4183658-supplementary.pdf]

Supplementary Material

1 Supplemental Figure S1

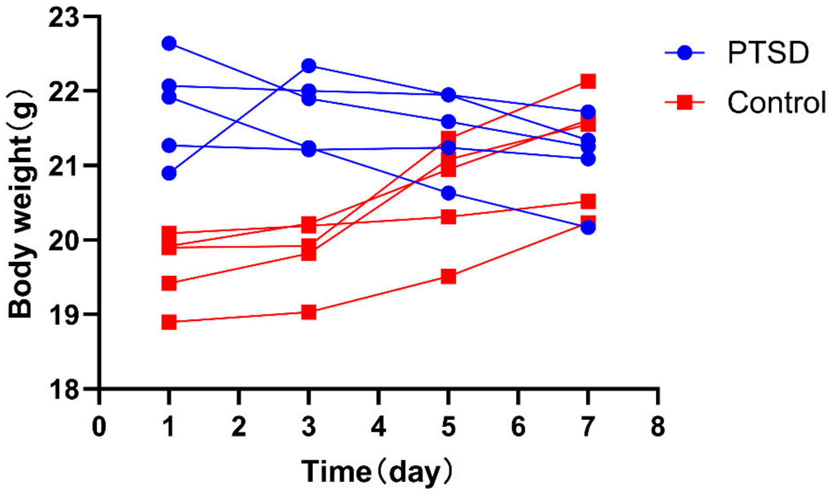

Supplementary Figure S1. Body weight changes of the experimental mice were recorded (n = 5).

2 Supplemental Figure S2

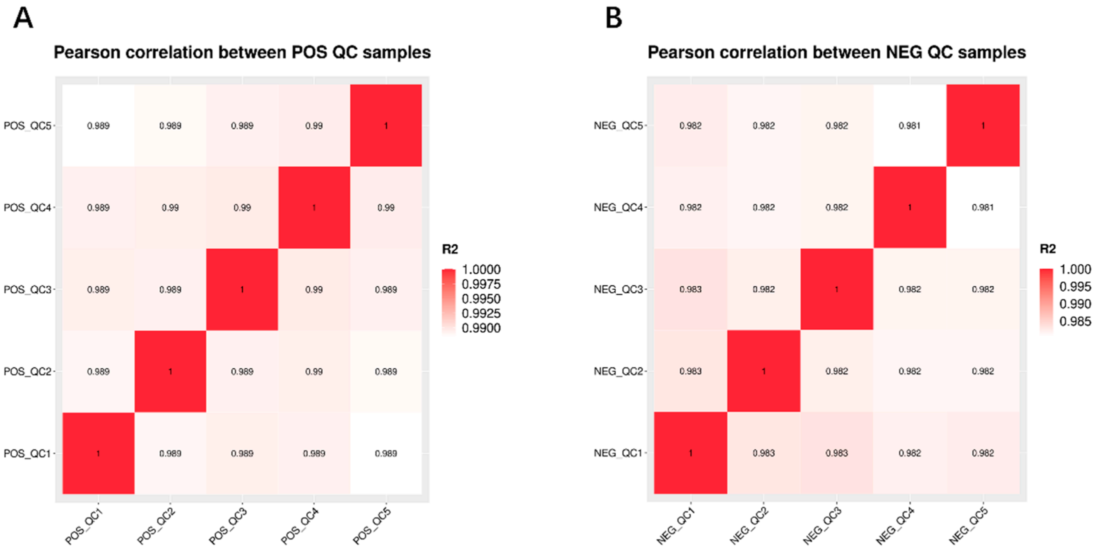

Supplementary Figure S2. The Pearson's correlation coefficient among QC samples. (A) positive ion mode; (B) Negative ion mode.

3 Supplemental Figure S3

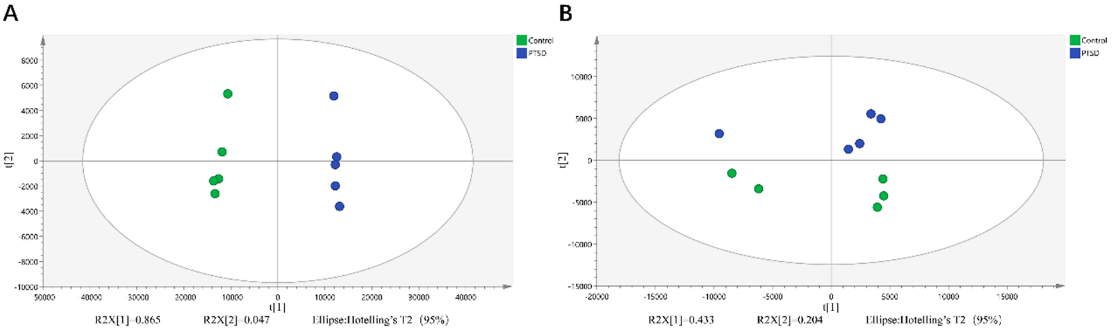

Supplementary Figure S3. PCA Results. (A) positive ion mode; (B) Negative ion mode.
